# Comparative Hepatoprotective Effects of Dapagliflozin and Trimetazidine in Diabetic Rats with Doxorubicin-Induced Liver Injury

**DOI:** 10.3390/biomedicines13112633

**Published:** 2025-10-27

**Authors:** Enver Ciftel, Omer Satiroglu, Muhammed Mursel Ogutveren, Tolga Mercantepe, Sibel Mataraci Karakas, Omer Genc, Adnan Yilmaz, Filiz Mercantepe

**Affiliations:** 1 Department of Endocrinology and Metabolism, Sivas Numune Hospital, 58060 Sivas, Türkiye; enver.ciftel@saglik.gov.tr; 2 Department of Cardiology, Faculty of Medicine, Recep Tayyip Erdogan University, 53100 Rize, Türkiye; 3 Department of Cardiology, Muş State Hospital, 49200 Muş, Türkiye; muhammedr4648@gmail.com; 4 Department of Histology and Embriology, Faculty of Medicine, Recep Tayyip Erdogan University, 53100 Rize, Türkiye; 5 Department of Biochemistry, Faculty of Medicine, Recep Tayyip Erdogan University, 53100 Rize, Türkiye; sibel.karakas@erdogan.edu.tr (S.M.K.); adnan.yilmaz@erdogan.edu.tr (A.Y.); 6 Department of Medical Oncology, Faculty of Medicine, Necmettin Erbakan University, 42090 Konya, Türkiye; omergenc58@hotmail.com; 7 Department of Endocrinology and Metabolism, Recep Tayyip Erdogan University, Training and Research Hospital, 53100 Rize, Türkiye; filiz.mercantepe@saglik.gov.tr

**Keywords:** dapagliflozin, diabetes mellitus, doxorubicin, liver injury, oxidative stress, trimetazidine

## Abstract

**Background:** Diabetes mellitus and cancer often coexist, increasing the risk of liver injury. Doxorubicin (DOXO) is a widely used antineoplastic drug with known hepatotoxic effects. Dapagliflozin (DAPA) and trimetazidine (TMZ) have been reported to exert hepatoprotective actions, but their combined effects remain unclear. **Methods:** Forty-eight male Sprague Dawley rats were allocated into six groups: control, streptozotocin (STZ), STZ + DOXO, STZ + DOXO + DAPA, STZ + DOXO + TMZ, and STZ + DOXO + DAPA + TMZ. Liver injury was assessed by histopathology, oxidative stress markers (MDA, GSH), and immunohistochemistry (Tumor Necrosis Factor-alpha (TNF-α), 8-Hydroxy-2′-deoxyguanosine (8-OHdG), Caspase-3, Transforming Growth Factor-beta 1 (TGF-β1), Terminal deoxynucleotidyl transferase dUTP nick end labeling (TUNEL), Nuclear Factor kappa-B/p65 (NF-κB/p65)). **Results:** STZ and STZ + doxorubicin groups developed marked hepatic injury. Unexpectedly, the STZ + doxorubicin group showed lower alanine transaminase (ALT) and aspartate aminotransferase (AST) levels, along with reduced Malondialdehyde (MDA) and elevated glutathione (GSH), suggesting compensatory antioxidant and apoptotic responses. Dapagliflozin more effectively normalized transaminases and reduced oxidative DNA damage, whereas trimetazidine exerted stronger effects on MDA, GSH, and inflammatory markers. The combination provided additive but not consistently superior benefits. Immunohistochemical analyses confirmed these findings, showing attenuated expression of TNF-α, 8-OHdG, caspase-3, and TGF-β1 and reduced TUNEL-positive hepatocytes and NF-κB/p65 immunoreactivity following treatment, indicating coordinated anti-apoptotic and anti-inflammatory effects. **Conclusions:** Dapagliflozin and trimetazidine each attenuated diabetes- and doxorubicin-related hepatic injury through partly distinct mechanisms, with the combination providing additive but not consistently superior effects. These findings suggest a potential hepatoprotective role for both agents; however, the clinical implications remain uncertain and require confirmation in further mechanistic and translational studies.

## 1. Introduction

Diabetes mellitus and cancer frequently coexist, and this comorbidity is of growing clinical importance. Epidemiological studies report that approximately 8–18% of patients with cancer also have diabetes, with prevalence varying by cancer type and population [1]. Recent large-scale studies have shown that nearly one-quarter (24.4%) of cancer patients in the United States have type 2 diabetes [2]. Conversely, diabetic patients are at higher risk of developing certain malignancies, including liver, pancreatic, colorectal, and breast cancers. The coexistence of diabetes and cancer not only worsens prognosis but also increases vulnerability to treatment-related toxicities. In particular, doxorubicin, a cornerstone anthracycline chemotherapy, is widely used in these populations, making diabetic patients more susceptible to drug-induced organ injury, including hepatic toxicity [3,4,5].

Doxorubicin is well known for its potent cytotoxic effects against various malignancies, including breast cancer, lymphomas, and sarcomas [3]. However, its clinical utility is significantly limited by serious toxicities, most notably cardiotoxicity and hepatotoxicity, which are primarily mediated through oxidative stress, inflammation, and apoptotic pathways [4,5]. Mechanistically, doxorubicin induces oxidative stress, inflammation, and apoptosis in hepatic tissue, leading to lipid peroxidation, depletion of glutathione (GSH), cytokine release, DNA damage, caspase activation, and eventually fibrosis. Experimental models have confirmed that doxorubicin increases hepatic malondialdehyde (MDA) levels, depletes GSH, and triggers caspase-3-mediated apoptosis in rat hepatocytes [6,7]. Simultaneously, streptozotocin (STZ)-induced diabetes in animal models mimics human type 1 diabetes by selectively destroying pancreatic β-cells, resulting in chronic hyperglycemia and associated liver damage [8,9]. These two conditions, whether acting independently or synergistically, may exacerbate hepatic injury via enhanced oxidative stress and inflammatory responses.

Dapagliflozin (DAPA), a sodium-glucose cotransporter 2 (SGLT2) inhibitor originally developed for glycemic control in type 2 diabetes, has demonstrated promising hepatoprotective effects in both experimental and clinical studies [10,11,12,13,14,15,16,17,18,19]. In models of diabetic steatohepatitis and liver injury induced by carbon tetrachloride (CCl_4_) or thioacetamide, DAPA was shown to alleviate hepatic inflammation and fibrosis by modulating oxidative stress, macrophage polarization, and various molecular pathways including Nuclear factor erythroid 2–related factor 2 / Heme oxygenase-1 (Nrf2/HO-1), Sirtuin 1 / AMP-activated protein kinase / Peroxisome proliferator-activated receptor gamma coactivator 1-alpha / Forkhead box protein O1 (Sirt1/AMPK/PGC-1α/FoxO1), and Toll-like receptor 4 / Transforming growth factor-beta 1 / Phosphoinositide 3-kinase (TLR4/TGF-β1/PI3K) [10,11,20,21,22,23]. Recent evidence also indicates that DAPA exerts protective effects against bile duct ligation-induced fibrosis through the suppression of fibrosis markers and proinflammatory cytokines [24]. In addition, trimetazidine (TMZ), a metabolic anti-ischemic agent, has also demonstrated cytoprotective effects in models of chemical liver injury, including hepatic ischemia–reperfusion and doxorubicin-induced hepatotoxicity, by attenuating oxidative stress, inflammation, and apoptotic signaling, particularly through modulation of caspase-3 activity [3,4,25,26,27,28].

Despite the available evidence on the separate hepatoprotective properties of dapagliflozin and trimetazidine, to the best of our knowledge, no previous study has directly compared their individual and combined effects in a model that mimics the complex triad of STZ-induced diabetes and doxorubicin-related hepatic injury. Liver damage due to diabetes may be further aggravated by doxorubicin through metabolic stress, oxidative imbalance, and impaired perfusion, making this combined model particularly relevant for exploring secondary hepatic injury. Addressing this gap, we sought to determine whether dapagliflozin and/or trimetazidine could mitigate liver injury by histopathological evaluation, biochemical markers of oxidative stress (MDA, GSH), and immunohistochemical (IHC) analysis of inflammation (Tumor necrosis factor-alpha (TNF-α), Nuclear factor kappa B p65 subunit (NF-κB/p65)), oxidative DNA damage (8-hydroxy-2′-deoxyguanosine; 8-OHdG), apoptosis (caspase-3, Terminal deoxynucleotidyl transferase dUTP nick end labeling (TUNEL)), and fibrosis (transforming growth factor-β1; TGF-β1).

We hypothesized that combining dapagliflozin and trimetazidine could provide complementary hepatoprotective effects due to their distinct mechanisms of action. While dapagliflozin predominantly exerts anti-inflammatory and antifibrotic effects through modulation of oxidative stress and metabolic pathways, trimetazidine primarily enhances mitochondrial energy efficiency and reduces oxidative damage by limiting fatty acid oxidation and attenuating caspase-3 mediated apoptosis. Therefore, their combination was expected to yield enhanced hepatoprotection in the setting of diabetes complicated by doxorubicin exposure.

Therefore, the present study aimed to evaluate the hepatoprotective potential of dapagliflozin and trimetazidine, both individually and in combination, in STZ-induced diabetic rats subjected to doxorubicin-induced cardiotoxic stress. To elucidate the underlying mechanisms of tissue protection and repair, histological, biochemical, and immunohistochemical analyses were conducted.

## 2. Materials and Methods

This experimental study was conducted using archived liver tissue samples obtained from a previously approved animal study that investigated the effects of dapagliflozin and trimetazidine on cardiac injury in diabetic rats with doxorubicin-induced cardiotoxicity [29]. The liver specimens were collected postmortem and preserved under optimal conditions for future analysis. In the present study, those preserved liver tissues were used for biochemical, histopathological, and immunohistochemical evaluations aimed at assessing hepatic injury and the potential protective roles of dapagliflozin and trimetazidine.

All procedures adhered strictly to ethical standards. The original experimental protocol had received approval from Recep Tayyip Erdogan University Animal Experiments Ethical Committee (Approval number: 2022/16 Approval Date: 26 April 2022). An additional amendment was granted to utilize archived liver tissues from that project for this hepatotoxicity-focused study. All experimental processes were conducted in accordance with the ARRIVE (Animal Research: Reporting of In Vivo Experiments) guidelines, ensuring animal welfare and scientific integrity throughout the study design and implementation [30].

### 2.1. Experimental Study

The experimental design of the study included a total of 48 male Sprague Dawley rats, aged 6–8 weeks and weighing approximately 210 ± 30 g. The animals were randomly assigned into six equal groups (*n* = 8 per group) as follows:

Control Group (Group 1): No intervention was applied. Rats were maintained under standard laboratory conditions and received a regular diet.

Diabetes Group (STZ) (Group 2): Diabetes was induced by a single intraperitoneal injection of streptozotocin (STZ) at a dose of 55–60 mg/kg. Blood glucose levels were measured using a glucometer (On Call Plus, Acon Laboratories Inc., 10125 Mesa Rim Road, San Diego, CA, USA) from the tail vein 72 h after injection. Rats with glucose levels >250 mg/dL were considered diabetic [31].

Diabetes + Doxorubicin Group (STZ + DOXO) (Group 3): Following diabetes induction with STZ as above, rats received intraperitoneal doxorubicin (5 mg/kg) once per week for four consecutive weeks (cumulative dose: 20 mg/kg) to induce cardiotoxicity [32].

Diabetes + Doxorubicin + Trimetazidine Group (STZ + DOXO + TMZ) (Group 4): After induction of diabetes and cardiotoxicity as in Group 3, rats were treated with oral trimetazidine (10 mg/kg/day) for 14 consecutive days, starting one week after the final doxorubicin dose [3].

Diabetes + Doxorubicin + Dapagliflozin Group (STZ + DOXO + DAPA) (Group 5): Following the same protocol for diabetes and cardiotoxicity induction, dapagliflozin was administered orally (10 mg/kg/day) for 14 consecutive days [24,33].

Diabetes + Doxorubicin + Trimetazidine + Dapagliflozin Group (STZ + DOXO + TMZ + DAPA) (Group 6): Rats received both trimetazidine (10 mg/kg/day) and dapagliflozin (10 mg/kg/day) for 14 days after the induction of diabetes and cardiotoxicity.

All animals were monitored daily for clinical signs and maintained under controlled environmental conditions (22 ± 2 °C, 12 h light/dark cycle, with ad libitum access to food and water). After diabetes induction with streptozotocin, doxorubicin was administered intraperitoneally once weekly for 4 consecutive weeks (cumulative dose 20 mg/kg). One week after the final doxorubicin dose, dapagliflozin (10 mg/kg/day, oral gavage) and/or trimetazidine (10 mg/kg/day, oral) treatments were initiated and continued for 14 days. Twenty-four hours after the last administration of dapagliflozin or trimetazidine, all animals were sacrificed under deep anesthesia using a combination of ketamine (50 mg/kg) and xylazine (10 mg/kg), and liver tissues were collected for histopathological, biochemical, and immunohistochemical analyses. The total experimental duration was 6 weeks following diabetes induction. Samples were divided for histopathological evaluation (fixed in 10% buffered formalin), immunohistochemical staining (TNF-α, 8-OHdG, caspase-3, TGF-β, TUNEL and NF-kβ/p65), and biochemical analyses including MDA and reduced GSH levels [34]. Histological and immunohistochemical evaluations were carried out by an experienced histopathologist blinded to group assignments. Liver damage was semi-quantitatively scored based on standard criteria, including hepatocellular degeneration, necrosis, inflammation, and sinusoidal congestion. Blood samples were collected via intracardiac puncture under sterile conditions at the end of the experimental period when rats were anesthetized. The blood was allowed to clot at room temperature and then centrifuged to obtain serum. Serum levels of alanine aminotransferase (ALT) and aspartate aminotransferase (AST) were measured by the calorimetric method, and the results were expressed in international units per liter (IU/L). All eight rats from each group were included in the biochemical, histopathological, and immunohistochemical analyses.

### 2.2. Study Outcomes

The primary outcomes of this study were to evaluate the degree of hepatic injury and assess the potential protective effects of dapagliflozin, trimetazidine, and their combination in diabetic rats exposed to doxorubicin-induced cardiotoxicity. Hepatoprotective effects were examined using biochemical analyses of serum transaminases (ALT, AST) and liver oxidative stress markers (MDA, GSH), histopathological evaluation of liver architecture (steatosis, necrosis, inflammation, sinusoidal congestion), immunohistochemical assessment of key markers related to inflammation (TNF-α), apoptosis (Caspase-3), oxidative DNA damage (8-OHdG), and fibrosis (TGF-β).

Secondary outcomes included comparison between monotherapy (dapagliflozin or trimetazidine) and combined therapy (dapagliflozin + trimetazidine) groups to determine synergistic effects on hepatic injury markers.

It should be noted that the liver tissues analyzed in the present study were originally obtained from an experiment primarily designed to investigate cardiac injury [29]. Although the preservation and handling procedures were performed under optimal conditions, the study was not specifically powered for hepatology-focused endpoints. Therefore, potential limitations such as sampling bias and reduced statistical sensitivity for liver-specific outcomes should be acknowledged.

### 2.3. Biochemical Analysis

#### Biochemical Analysis of Liver Tissue

Liver tissues were homogenized in a buffer containing 20 mM sodium phosphate and 140 mM potassium chloride (pH 7.4) at a ratio of 1:9 (tissue weight [g]: buffer volume [mL]) using a QIAGEN TissueLyser II [35]. The homogenates were centrifuged at +4 °C, 800× *g* for 10 min, and the resulting supernatants were used for biochemical analyses. Lipid peroxidation was assessed by measuring MDA levels using a modified thiobarbituric acid reactive substances (TBARS) assay, with the pink-colored complex read at 532 nm. Reduced GSH levels, representing antioxidant capacity, were determined spectrophotometrically based on the reaction with 5,5′-dithiobis-(2-nitrobenzoic acid) (DTNB), and absorbance was measured at 412 nm. Total thiol content was also evaluated using DTNB, and the yellow-colored complex was measured at the same wavelength. Concentrations were calculated using standard curves and expressed as nmol/g tissue for MDA, and mM/g tissue for total thiol groups [36,37].

### 2.4. Histopathological Evaluation

Liver tissues were fixed in 10% neutral buffered formalin, embedded in paraffin, sectioned at 4–5 µm using a rotary microtome (Leica RM2525, Leica, Nussloch, Germany) and stained with Harris hematoxylin and eosin (H&E). Histological and immunohistochemical images were obtained using an Olympus BX51 TF trinocular light microscope (Olympus, Tokyo, Japan) equipped with an Olympus DP74 digital camera. Images were captured under standardized conditions, and scale bars were automatically added to all micrographs to accompany magnification information. The histopathological evaluation was performed by a blinded histopathologist using a semi-quantitative scoring system. The parameters assessed included intralobular degeneration and focal necrosis, periportal bridging necrosis, steatotic hepatocytes (presence of cytoplasmic lipid vacuoles), and vascular congestion. Each parameter was scored from 0 to 4 according to the extent of involvement as follows: 0 = involvement in ≤5% of the tissue, 1 = 6–25%, 2 = 26–50%, 3 = 51–75%, and 4 = >76% of the tissue [27,38].

### 2.5. Immunohistochemical Evaluation

Immunohistochemical analysis was performed to assess inflammation, oxidative DNA damage, apoptosis, and fibrosis in liver tissues. Sections (1–2 µm thick) were deparaffinized, rehydrated, and subjected to antigen retrieval using citrate buffer (pH 6.0) in a microwave oven. Endogenous peroxidase activity was blocked with 3% hydrogen peroxide. After blocking nonspecific binding sites, the following primary antibodies were applied: TNF-α (1/100, ab220210, Abcam, Cambridge, UK), NF-kβ/p65 (1/200, BT-AP05991, Shanghai Korain Biotech, Shanghai, China) to evaluate inflammatory response, 8-OHdG (1/200, sc-66036, Santa Cruz Biotechnology, Inc., Dallas, TX, USA) to assess oxidative DNA damage, Caspase-3 (1/100, ab4051, Abcam, Cambridge, UK) and TUNEL (ab206386, Abcam, Cambridge, UK) as a marker of apoptosis, TGF-β1 (1/200, ab64715, Abcam, Cambridge, UK) to detect fibrotic and remodeling activity. All primary antibodies were validated by the manufacturers for immunohistochemistry in rat tissues. The specificity of the antibodies was further supported by previous publications using the same clones [39,40,41,42]. In our study, negative controls (omission of primary antibody and isotype controls) showed no staining, confirming specificity.

All primary antibodies were incubated overnight at 4 °C. After rinsing, tissue sections were incubated with appropriate biotinylated secondary antibodies (Goat Anti-Rabbit IgG H&L (HRP), ab205718, Abcam, Cambridge, UK), followed by treatment with streptavidin–peroxidase complex. The chromogen 3,3′-diaminobenzidine (DAB) was used for visualization, and sections were counterstained with hematoxylin. Slides were examined under a light microscope. Immunopositivity was evaluated semi-quantitatively using a 5-point scoring system based on the percentage of positively stained cells: 0 = none (<5%), 1 = mild (6–25%), 2 = moderate (26–50%), 3 = severe (51–75%), and 4 = very severe (>76%) [27,43].

For each animal, five liver sections were examined, and 10 non-overlapping fields per section were evaluated at 40× magnification. Representative images shown in the figures were selected as typical examples corresponding to the median score of each group.

### 2.6. Statistical Analysis

All statistical analyses were performed using SPSS software version 25.0 (IBM Corp., Armonk, NY, USA). The normality of data distribution was assessed using the Shapiro–Wilk test, and homogeneity of variances was evaluated with Levene’s test. Parametric data are expressed as mean ± standard deviation (SD), while non-parametric data are presented as median (interquartile range, IQR).

For normally distributed continuous variables with homogeneous variances (e.g., MDA, GSH, ALT, AST), differences among groups were analyzed using one-way analysis of variance (ANOVA), followed by Tamhane’s T2 post hoc test in cases of variance heterogeneity. Effect sizes (eta-squared, η^2^) and corresponding Cohen’s f values were calculated, and post hoc power analyses were conducted to ensure sufficient statistical strength.

For non-normally distributed or ordinal data, such as histopathological and immunohistochemical scores, comparisons between groups were made using the Kruskal–Wallis test, followed by pairwise comparisons with Bonferroni-adjusted Dunn’s tests when appropriate. A *p*-value of <0.05 was considered statistically significant.

Our design is a 6-group one-way ANOVA (k = 6). Using the resource-equation approach for animal studies (acceptable error degrees of freedom, DF, 10–20), the required per-group size is *n*_min_ = 10/k + 1 = 10/6 + 1 ≈ 3 and *n*_max_ = 20/*k* + 1 = 20/6 + 1 ≈ 4. Thus, the recommended range is 3–4 rats/group (total N = 18–24). We used 8 rats/group (N = 48), which exceeds the minimum needed by this approach and therefore provides ample precision for hepatic endpoints [34,44].

For completeness, DF in our study is *k* (*n* − 1) = 6 × (8 − 1) = 42; exceeding the DF upper bound reflects conservative sampling rather than a validity concern in this context. The resource equation sets sufficiency thresholds to avoid under- or over-use, not hard validity limits [44].

## 3. Results

### 3.1. Biochemical Results

As shown in Table 1 and Table 2, comparative analysis of the six experimental groups revealed significant differences in all four biochemical parameters analyzed: MDA, GSH, AST, and ALT. Levene’s test was used to assess the homogeneity of variances across groups. The results indicated that variance was homogeneous only for AST (*p* > 0.05), while MDA, GSH, and ALT showed significant heterogeneity (*p* < 0.05). Accordingly, Tamhane’s T2 test was applied for pairwise comparisons of parameters with unequal variances, and Bonferroni correction was used for AST. Given the relatively small sample size (*n* = 8 per group), which may limit the sensitivity of conventional post hoc analyses, additional effect size (η^2^) and post hoc power calculations were performed to support the interpretation of findings. In all cases, the power values exceeded 0.90, indicating sufficient sensitivity to detect true group differences (Table 3).

Malondialdehyde (MDA) levels showed a statistically significant difference among the experimental groups (F(5, 42) = 3.610, *p* = 0.008), with a large effect size (η^2^ = 0.301), suggesting that 30.1% of the variance was attributable to group differences. Although the pairwise comparisons using Tamhane’s T2 test did not consistently yield statistically significant differences, post hoc power analysis yielded a value of 0.928, supporting the presence of a genuine group effect. MDA levels were significantly elevated in the STZ group compared to controls (78.88 ± 17.2 vs. 62.30 ± 10.3 nmol/g, *p* < 0.05), indicating increased lipid peroxidation. Interestingly, the STZ + DOXO group showed slightly lower MDA levels (67.36 ± 11.5), and both trimetazidine (55.40 ± 11.8) and combination therapy (62.37 ± 2.7) reduced MDA values even further. The combination group’s MDA level was comparable to that of the control group.

Glutathione (GSH) levels also differed significantly among the groups (F(5, 42) = 3.918, *p* = 0.005), with an effect size of η^2^ = 0.318, indicating that 31.8% of the variability in GSH concentration was explained by group membership. The statistical power was calculated as 0.948. GSH levels were significantly reduced in the STZ group (6.51 ± 1.3) compared to the control group (7.43 ± 1.0, *p* < 0.05), confirming oxidative stress-induced depletion. However, an unexpected moderate increase in GSH was observed in the STZ + DOXO group (7.61 ± 1.4), suggesting a possible compensatory upregulation of endogenous antioxidant mechanisms. Treatment with trimetazidine (10.02 ± 3.0) or combination therapy (7.23 ± 2.0) significantly elevated GSH levels, with the trimetazidine group showing the highest increase.

Aspartate aminotransferase (AST) levels demonstrated a highly significant group effect (F(5, 42) = 20.041, *p* < 0.001), with a very large effect size (η^2^ = 0.705). Post hoc power analysis yielded 1.000. One-way ANOVA revealed significant differences in AST levels among the experimental groups (*p* < 0.05). To identify the specific group differences, post hoc pairwise comparisons using the Bonferroni correction were conducted. The results are presented in Table 4. Bonferroni-adjusted pairwise comparisons revealed that AST levels were markedly elevated in the STZ (485 ± 120) and STZ + DOXO (203 ± 83) groups compared to controls (157 ± 36, *p* < 0.001). Group 2 (STZ) had significantly higher AST levels than Groups 1, 3, 5, and 6, while Group 4 (STZ + DOXO + TMZ) also demonstrated significantly higher AST than the same comparator groups. Dapagliflozin (Group 5) and combination therapy (Group 6) reduced AST levels to near-control values.

Alanine aminotransferase (ALT) levels were also significantly affected by the experimental conditions (F(5, 42) = 13.796, *p* < 0.001), with an eta-squared of 0.622. The post hoc power was 1.000. ALT levels were markedly elevated in both the STZ (201 ± 78) and STZ + DOXO (104 ± 44) groups compared to the control group (65 ± 9, *p* < 0.001). While trimetazidine did not significantly improve ALT levels (Group 4: 188 ± 42), dapagliflozin (64 ± 17) and combination therapy (92 ± 51) effectively reduced ALT concentrations, with dapagliflozin being particularly effective in restoring ALT to near-baseline levels.

### 3.2. Histopathological Results

**Figure 1:**
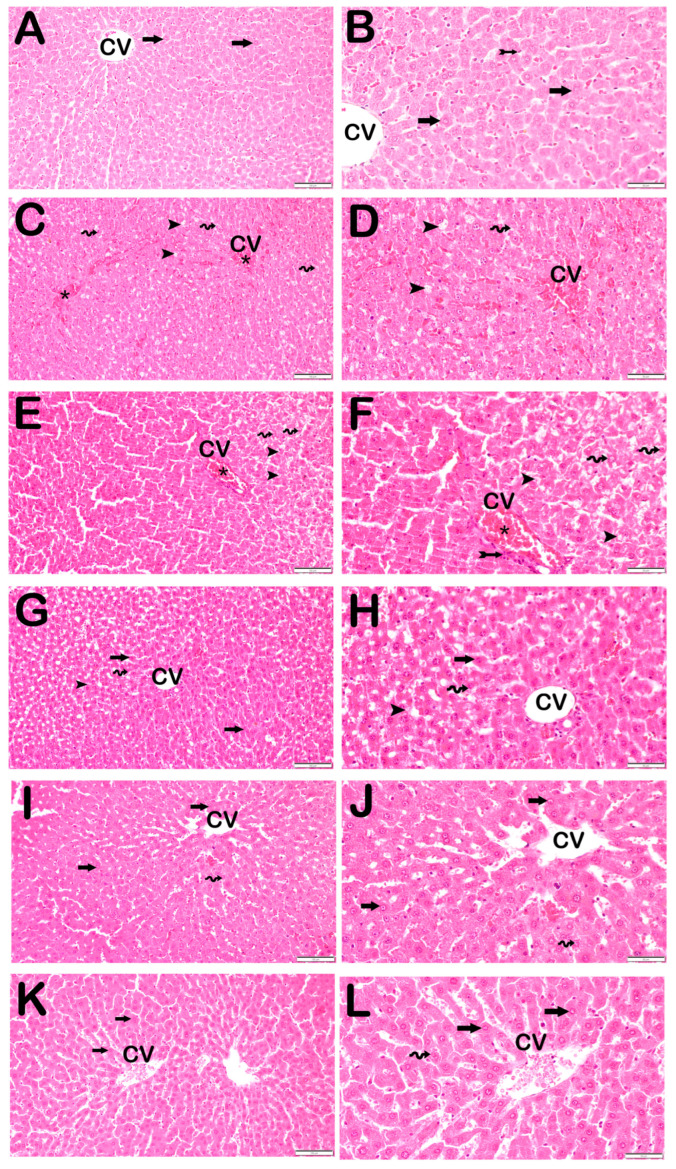
Representative light microscopic image of liver tissue sections stained with H&E. Central Ven (CV). For each marker, liver sections from eight rats per group (total N = 48) were analyzed. Each rat contributed five sections (8 × 6 groups × 5 = 240 sections in total), and 10 non-overlapping microscopic fields per section were evaluated at 20× and 40× magnification. (**A**) (×20), (**B**) (×40): Control group. Normal hepatocytes arranged in hepatic cords (arrow) and Kupffer cells within the sinusoids (tailed arrow) are observed. (LDS score: 0 (0–0)). (**C**) (×20), (**D**) (×40): STZ group. Necrotic hepatocytes with intralobular distribution and steatotic hepatocytes (spiral arrow) containing cytoplasmic lipid vacuoles (arrowhead) are evident, accompanied by widespread vascular congestion (asteriks). (LDS score: 8 (8–9)). (**E**) (×20), (**F**) (×40): STZ + DOXO group. Periportal (bridging necrosis) and intralobular necrosis (tailed arrow) are observed along with steatotic hepatocytes (spiral arrow) containing cytoplasmic lipid vacuoles (arrowhead) and vascular congestion (asteriks). (LDS score: 10 (9–11)). (**G**) (×20), (**H**) (×40): STZ + DOXO + TMZ group. Normal hepatocytes are arranged in hepatic cords (arrow). Reduced necrotic hepatocytes (arrowhead) and vascular congestion are noted. The number of steatotic hepatocytes (spiral arrow) has also decreased. (LDS score: 3 (2–3)). (**I**) (×20), (**J**) (×40): STZ + DOXO + DAPA group. Normal hepatocytes are arranged in hepatic cords (arrow). A decrease in necrotic hepatocytes (spiral arrow) is observed in both intralobular and periportal areas. (LDS score: 3 (3–4)). (**K**) (×20), (**L**) (×40): STZ + DOXO + TMZ + DAPA group. Normal hepatocytes are arranged in hepatic cords (arrow). A marked reduction is seen in both necrotic and steatotic hepatocytes (spiral arrow). (LDS score: 2.5 (2–3)).

As summarized in Table 5 and illustrated in Figure 1, the total histopathological liver damage score (LDS) was significantly increased in the STZ and STZ + DOXO groups (8 (8–9) and 10 (9–11), respectively) compared to the control group (0 (0–0), *p* < 0.001).

Histological features such as intralobular degeneration, periportal bridging necrosis, steatosis, and vascular congestion were markedly evident in these groups.

Trimetazidine and dapagliflozin treatments significantly improved all histopathological parameters, with combined therapy showing the greatest improvement, reducing the LDS to 2.5 (2–3) (*p* < 0.001 vs. STZ + DOXO group).

### 3.3. Immunohistochemical Results

**Figure 2:**
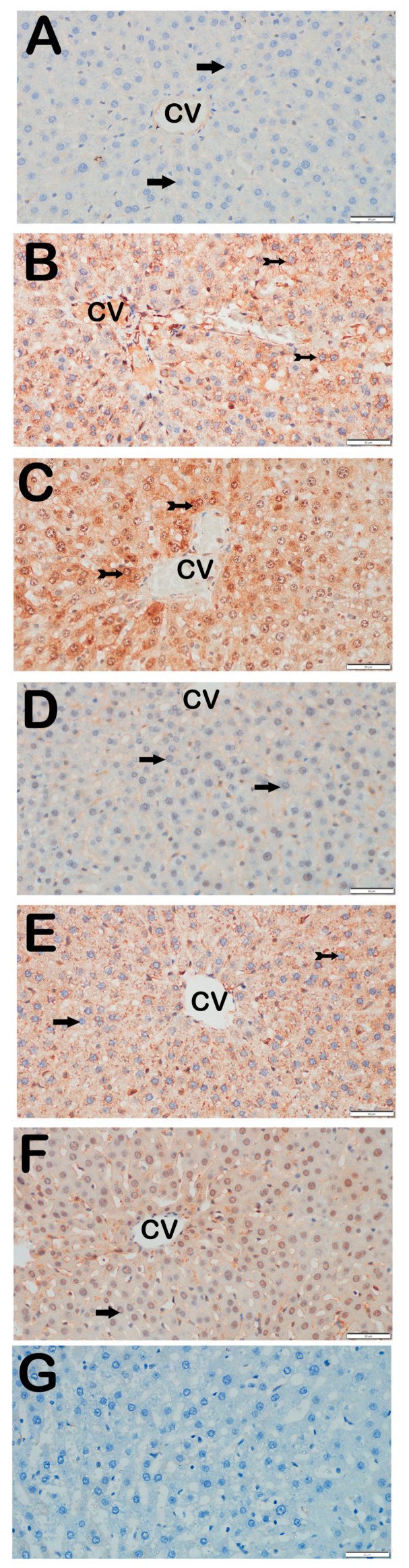
Representative light microscopic images of liver tissue sections stained with Caspase-3 primary antibody. For each marker, liver sections from eight rats per group (total N = 48) were analyzed. Each rat contributed five sections (8 × 6 groups × 5 = 240 sections in total), and 10 non-overlapping microscopic fields per section were evaluated at 40× magnification. (**A**) (×40): Control group. Hepatocytes in hepatic cords are immunonegative for Caspase-3 (arrow). (Caspase-3 positivity score: 0 (0–0)). (**B**) (×40): STZ group. Numerous apoptotic hepatocytes showing strong Caspase-3 positivity are observed in hepatic cords (tailed arrow). (Caspase-3 score: 2 (2–2)). (**C**) (×40): STZ + DOXO group. Increased number of hepatocytes with strong Caspase-3 positivity is evident (tailed arrow). (Caspase-3 score: 3 (2–3)). (**D**) (×40): STZ + DOXO + TMZ group. A reduction in Caspase-3-positive apoptotic hepatocytes is observed in hepatic cords (arrow). (Caspase-3 score: 1 (0–1)). (**E**) (×40): STZ + DOXO + DAPA group. The number of Caspase-3-positive hepatocytes decreases (tailed arrow). Hepatocytes in hepatic cords are immunonegative for Caspase-3 (arrow). (Caspase-3 score: 1 (0–1)). (**F**) (×40): STZ + DOXO + TMZ + DAPA group. A marked decrease in Caspase-3-positive apoptotic hepatocytes is noted in hepatic cords (arrow). (Caspase-3 score: 1 (1–1)). (**G**) (×40): Negative control sections processed in parallel without primary antibody showed no specific staining, confirming the specificity of the immunohistochemical procedure. *n* = 8 rats per group.

**Figure 3:**
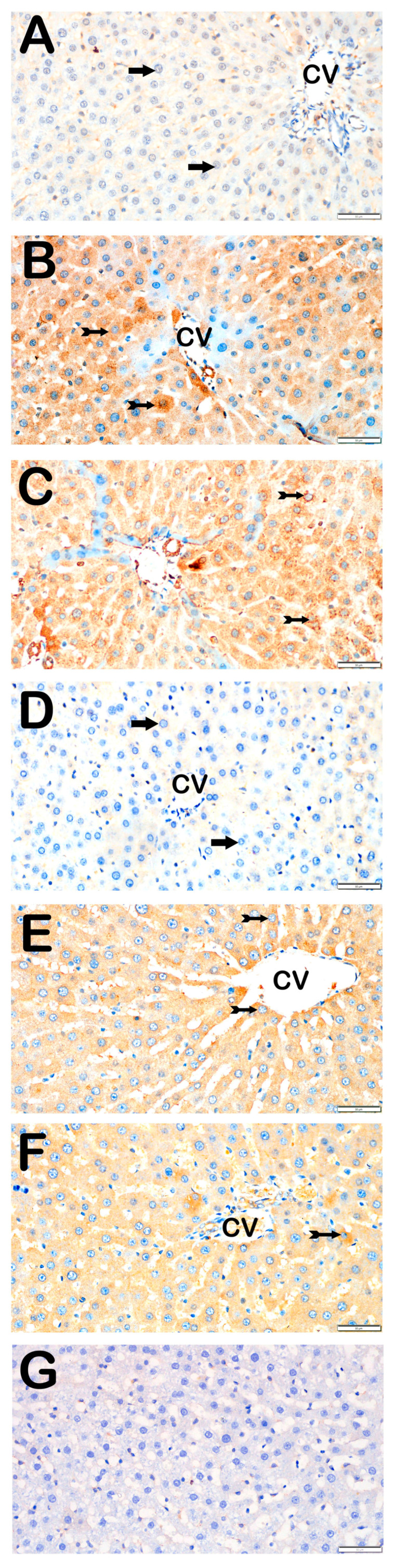
Representative light microscopic images of liver tissue sections stained with 8-OHdG primary antibody. For each marker, liver sections from eight rats per group (total N = 48) were analyzed. Each rat contributed five sections (8 × 6 groups × 5 = 240 sections in total), and 10 non-overlapping microscopic fields per section were evaluated at 40× magnification. (**A**) (×40): Control group. Normal hepatocytes are immunonegative for 8-OHdG (arrow). (8-OHdG positivity score: 0 (0–0)). (**B**) (×40): STZ group. Numerous hepatocytes showing strong 8-OHdG positivity are observed in hepatic cords (tailed arrow). (8-OHdG score: 2 (2–3)). (**C**) (×40): STZ+DOXO group. A marked increase in 8-OHdG-positive hepatocytes is evident (tailed arrow). (8-OHdG score: 3 (2–3)). (**D**) (×40): STZ+DOXO+TMZ group. A reduction in 8-OHdG-positive hepatocytes is observed in intralobular and perilobular regions (arrow). (8-OHdG score: 1 (1–1)). (**E**) (×40): STZ+DOXO+DAPA group. Decreased number of hepatocytes with 8-OHdG immunopositivity is observed (tailed arrow). (8-OHdG score: 1 (1–2)). (**F**) (×40): STZ + DOXO + TMZ + DAPA group. A noticeable reduction in 8-OHdG-positive hepatocytes is seen in hepatic cords (tailed arrow). (8-OHdG score: 2 (1–2)). (**G**) (×40): Negative control sections processed in parallel without primary antibody showed no specific staining, confirming the specificity of the immunohistochemical procedure. *n* = 8 rats per group.

**Figure 4:**
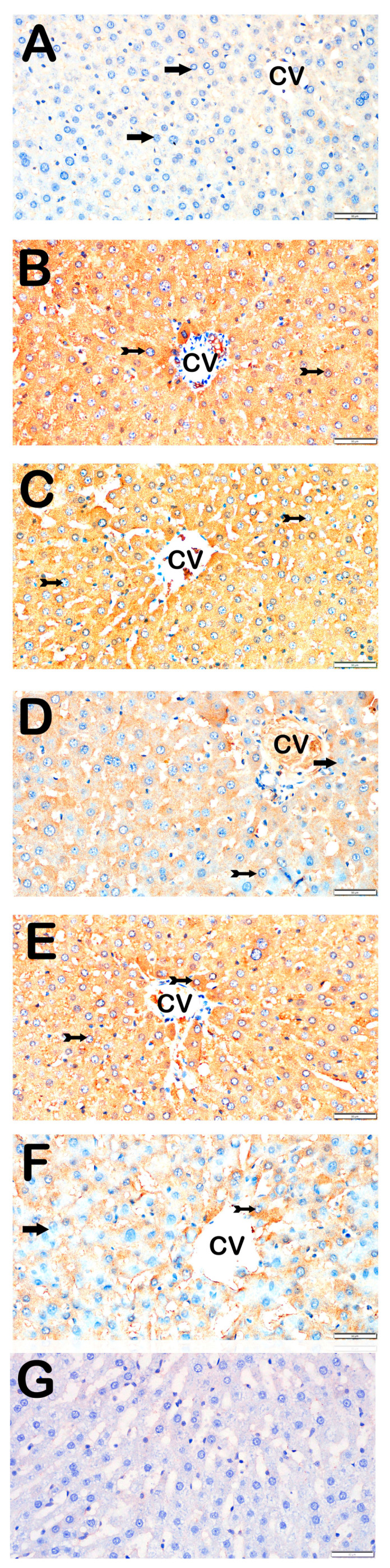
Representative light microscopic images of liver tissue sections stained with TNF-α primary antibody. For each marker, liver sections from eight rats per group (total N = 48) were analyzed. Each rat contributed five sections (8 × 6 groups × 5 = 240 sections in total), and 10 non-overlapping microscopic fields per section were evaluated at 40× magnification. (**A**) (×40): Control group. Hepatocytes in hepatic cords are immunonegative for TNF-α (arrow). (TNF-α positivity score: 0 (0–0)). (**B**) (×40): STZ group. Hepatocytes with strong TNF-α positivity are observed in hepatic cords (tailed arrow). (TNF-α score: 2 (2–3)). (**C**) (×40): STZ+DOXO group. A marked increase in TNF-α-positive hepatocytes is observed (tailed arrow). (TNF-α score: 3 (2–3)). (**D**) (×40): STZ + DOXO + TMZ group. The number of TNF-α-positive hepatocytes is reduced (tailed arrow). Hepatocytes in hepatic cords are immunonegative for TNF-α (arrow). (TNF-α score: 1 (1–2)). (**E**) (×40): STZ + DOXO + DAPA group. Decreased TNF-α-positive hepatocytes are observed in intra- and perilobular areas (tailed arrow). (TNF-α score: 2 (2–3)). (**F**) (×40): STZ + DOXO + TMZ + DAPA group. A reduction in TNF-α-positive hepatocytes is noted in hepatic cords (tailed arrow). Hepatocytes in hepatic cords are immunonegative for TNF-α (arrow). (TNF-α score: 2 (1–2)). (**G**) (×40): Negative control sections processed in parallel without primary antibody showed no specific staining, confirming the specificity of the immunohistochemical procedure. *n* = 8 rats per group.

**Figure 5:**
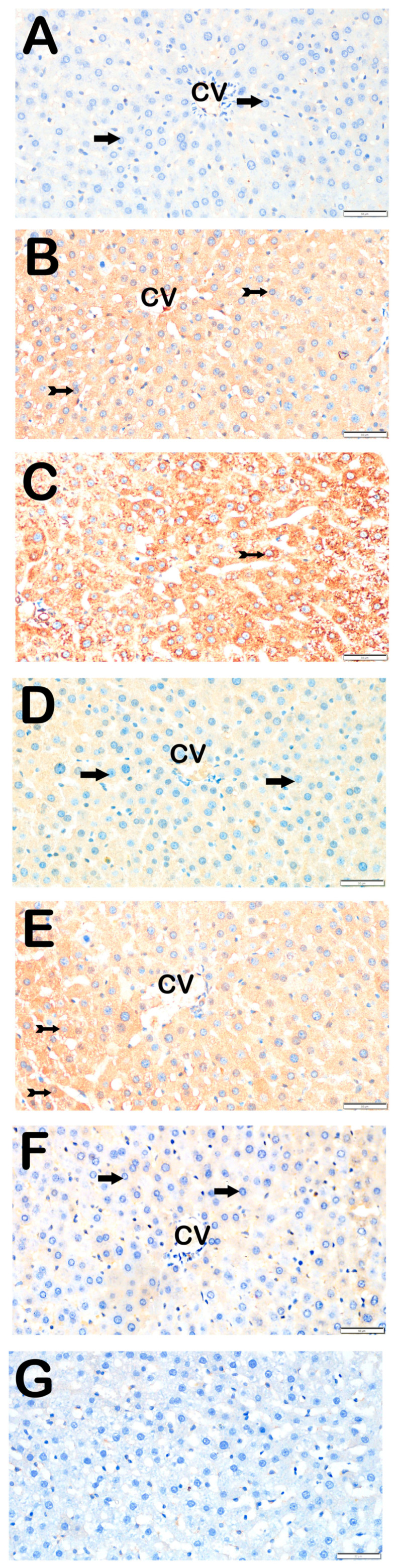
Representative light microscopic images of liver tissue sections incubated with TGF-β1 primary antibody. For each marker, liver sections from eight rats per group (total N = 48) were analyzed. Each rat contributed five sections (8 × 6 groups × 5 = 240 sections in total), and 10 non-overlapping microscopic fields per section were evaluated at 40× magnification. (**A**) (×40): Control group. Hepatocytes in hepatic cords are immunonegative for TGF-β1 (arrow). (TGF-β1 positivity score: 0 (0–0)). (**B**) (×40): STZ group. Numerous hepatocytes showing strong TGF-β1 positivity are observed in hepatic cords (tailed arrow). (TGF-β1 score: 2 (1–2)). (**C**) (×40): STZ + DOXO group. Increased number of TGF-β1-positive hepatocytes is evident in intra- and perilobular areas (tailed arrow). (TGF-β1 score: 2 (2–3)). (**D**) (×40): STZ + DOXO + TMZ group. A marked reduction in TGF-β1-positive hepatocytes is seen, especially in perilobular regions (arrow). (TGF-β1 score: 1 (0–1)). (**E**) (×40): STZ + DOXO + DAPA group. TGF-β1-positive hepatocytes are reduced in intra- and perilobular areas (tailed arrow). (TGF-β1 score: 2 (1–2)). (**F**) (×40): STZ + DOXO + TMZ + DAPA group. A significant decrease in TGF-β1-positive hepatocytes is observed in hepatic cords (arrow). (TGF-β1 score: 0.5 (0–1)). (**G**) (×40): Negative control sections processed in parallel without primary antibody showed no specific staining, confirming the specificity of the immunohistochemical procedure. *n* = 8 rats per group.

**Figure 6:**
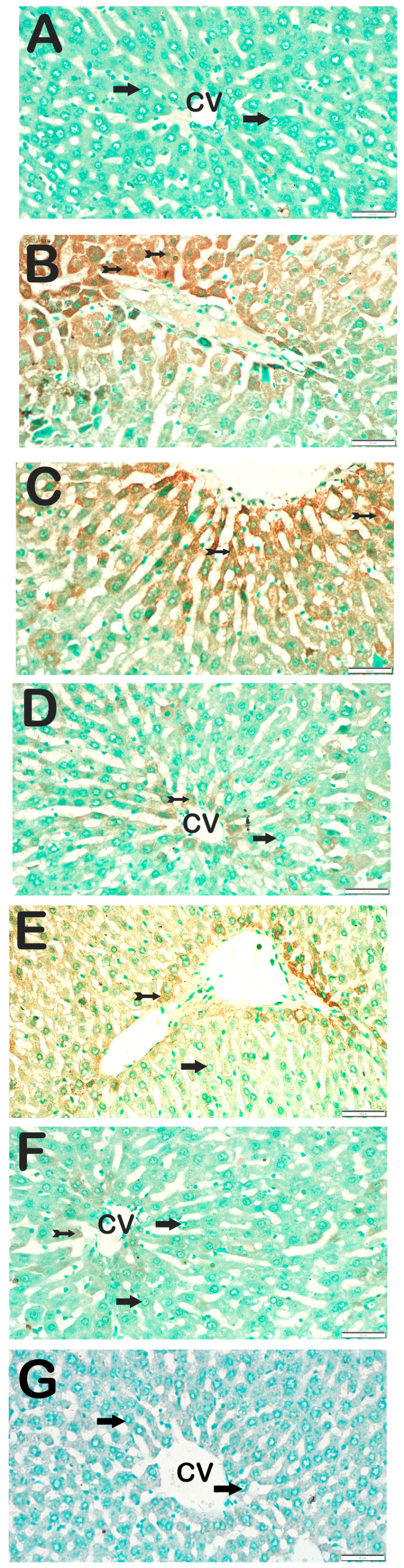
Representative light microscopic images of liver tissue sections showing apoptotic hepatocytes detected by the terminal deoxynucleotidyl transferase dUTP nick-end labeling (TUNEL) method. For each marker, liver sections from eight rats per group (total N = 48) were analyzed. Each rat contributed five sections (8 × 6 groups × 5 = 240 sections in total), and 10 non-overlapping microscopic fields per section were evaluated at 40× magnification. (**A**) (×40): Control group. Hepatocytes within the hepatic cords exhibit normal morphology and are immunonegative for TUNEL (arrow). (TUNEL positivity score: 0 (0–0)). (**B**) (×40): STZ group. Numerous apoptotic hepatocytes are observed within the hepatic cords of liver tissue (tailed arrow). (TUNEL score: 2 (2–2)). (**C**) (×40): STZ+DOXO group. A marked increase in the number of apoptotic hepatocytes is evident within the hepatic cords (tailed arrow). (TUNEL score: 3 (2–3)). (**D**) (×40): STZ + DOXO + SGLT2 group. A reduction in the number of apoptotic hepatocytes within the hepatic cords is observed (tailed arrow). Hepatocytes within the hepatic cords exhibit normal morphology and are immunonegative for TUNEL (arrow). (TUNEL positivity score: 1 (0–1)). (**E**) (×40): STZ + DOXO + TMZ group. Fewer apoptotic hepatocytes are seen within the hepatic cords of liver tissue (tailed arrow). Hepatocytes within the hepatic cords exhibit normal morphology and are immunonegative for TUNEL (arrow). (TUNEL positivity score: 1 (0–2)). (**F**) (×40): STZ + DOXO + SGLT2 + TMZ group. A noticeable decrease in apoptotic hepatocytes within the hepatic cords is evident (tailed arrow). Hepatocytes within the hepatic cords exhibit normal morphology and are immunonegative for TUNEL (arrow). (TUNEL positivity score: 1 (0–1)). (**G**) (×40): Negative control sections processed in parallel without primary antibody showed no specific staining (arrow), confirming the specificity of the immunohistochemical procedure. *n* = 8 rats per group.

**Figure 7:**
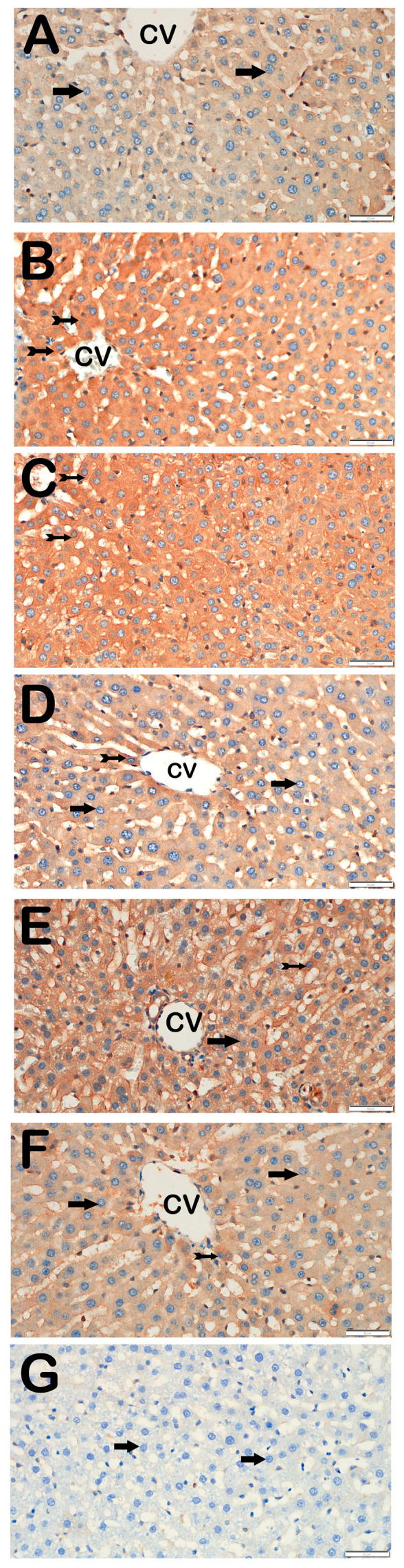
Representative light microscopic images of liver tissue sections incubated with NF-κB/p65 primary antibody. For each marker, liver sections from eight rats per group (total N = 48) were analyzed. Each rat contributed five sections (8 × 6 groups × 5 = 240 sections in total), and 10 non-overlapping microscopic fields per section were evaluated at 40× magnification. (**A**) (×40): Control group. Hepatocytes within the hepatic cords exhibit normal morphology and are immunonegative for NF-κB/p65 (arrow). (NF-κB/p65 positivity score: 0 (0–0)). (**B**) (×40): STZ group. Numerous hepatocytes showing strong immunopositivity for NF-κB/p65 are observed in liver tissue (tailed arrow). (NF-κB/p65 score: 2 (2–2)). (**C**) (×40): STZ + DOXO group. A marked increase in hepatocytes exhibiting strong NF-κB/p65 immunopositivity is evident within the hepatic cords (tailed arrow). (NF-κB/p65 score: 3 (2–3)). (**D**) (×40): STZ + DOXO + DAPA group. A noticeable decrease in the number of hepatocytes showing NF-κB/p65 immunopositivity is observed (tailed arrow). Hepatocytes within the hepatic cords exhibit normal morphology and are immunonegative for NF-κB/p65 (arrow). (NF-κB/p65 positivity score: 1 (1–1)). (**E**) (×40): STZ + DOXO + TMZ group. A reduced number of NF-κB/p65-positive hepatocytes is observed in intra- and perilobular areas (tailed arrow). Hepatocytes within the hepatic cords exhibit normal morphology and are immunonegative for NF-κB/p65 (arrow). (NF-κB/p65 positivity score: 1 (1–2)). (**F**) (×40): STZ + DOXO + DAPA + TMZ group. A decrease in hepatocytes showing NF-κB/p65 immunopositivity within the hepatic cords is observed (tailed arrow). Hepatocytes within the hepatic cords exhibit normal morphology and are immunonegative for NF-κB/p65 (arrow). (NF-κB/p65 positivity score: 1 (0–1)). (**G**) (×40): Negative control sections processed in parallel without primary antibody showed no specific staining (arrow), confirming the specificity of the immunohistochemical procedure. *n* = 8 rats per group.

Immunohistochemical positivity scores for Caspase-3, 8-OHdG, TNF-α, TGF-β1, NF-kβ/p65, and TUNEL are presented in Table 6 and Figure 2, Figure 3, Figure 4, Figure 5, Figure 6 and Figure 7. All six markers were significantly elevated in the STZ and STZ + DOXO groups compared to controls (*p* < 0.05), confirming enhanced apoptosis, oxidative DNA damage, inflammation, and fibrosis.

Trimetazidine and dapagliflozin, both alone and in combination, significantly reduced positivity scores of Caspase-3 (from 3 (2–3) to 1 (0–1)), 8-OHdG (from 3 (2–3) to 1 (1–2)), TNF-α (from 3 (2–3) to 1 (1–2)), TGF-β1 (from 2 (2–3) to 0.5 (0–1)), NF-κB/p65 (from 3 (2–3) to 1 (0–1)), and TUNEL (from 3 (2–3) to 1 (0–1); *p* < 0.05). The greatest reductions were observed in the combined therapy group, particularly for Caspase-3, TGF-β1, and NF-κB/p65 (*p* < 0.001 vs. STZ + DOXO).

## 4. Discussion

This study examined whether dapagliflozin, trimetazidine, or their combination mitigates hepatic injury in STZ–diabetic rats exposed to doxorubicin. Across endpoints, both agents attenuated injury; DAPA more consistently improved transaminases and reduced apoptosis/oxidative DNA damage, whereas TMZ showed stronger antioxidant modulation. The combination produced additional structural (histological) benefits but did not surpass DAPA for biochemical outcomes, supporting a partially complementary—rather than synergistic—interaction.

DOXO is known to promote ROS generation and deplete antioxidant defenses [3,4,5]. As expected, MDA levels increased and GSH levels decreased in the diabetic group. Interestingly, despite the expected oxidative stress burden, the STZ + DOXO group demonstrated reduced MDA levels and increased GSH compared to the STZ-only group. This paradoxical finding may be explained by compensatory activation of endogenous antioxidant pathways. Doxorubicin has been reported to induce nuclear factor erythroid 2-related factor 2 (Nrf2) signaling, which enhances glutathione synthesis and upregulates antioxidant enzymes under certain conditions [45,46,47,48]. Such an adaptive response could account for the observed elevation in GSH and concomitant reduction in lipid peroxidation markers [6,7]. In addition, the timing of sample collection may have influenced the results, as transient spikes in MDA during the acute phase of oxidative stress might have declined by the time of tissue harvest. Therefore, the lower MDA and higher GSH levels in the STZ + DOXO group should be interpreted as reflecting compensatory and time-dependent antioxidant responses, rather than a true reduction in hepatocellular injury.

Among the treatment groups, TMZ produced stronger direct antioxidant effects, lower MDA and higher GSH than DAPA, consistent with its roles in preserving mitochondrial function, reducing fatty acid oxidation, and limiting oxidative bursts [49]. Although DAPA is linked mechanistically to AMPK/Nrf2-driven GSH synthesis [21], GSH increases were modest, possibly due to pharmacokinetic or indirect systemic factors. The combination did not add to these antioxidant gains, suggesting a ceiling effect or feedback that may limit excessive Nrf2 activation.

Interestingly, the STZ + DOXO group showed paradoxical biochemical findings, including lower ALT and AST levels and reduced MDA alongside increased GSH, despite clear histopathological and immunohistochemical evidence of liver injury. One possible explanation is that doxorubicin may preferentially induce apoptotic rather than necrotic cell death, thereby limiting membrane rupture, transaminase leakage, and lipid peroxidation. In contrast, STZ-induced hyperglycemia promotes hepatocellular necrosis, oxidative stress, and enzyme release. Compensatory antioxidant responses, potentially involving Nrf2-mediated glutathione upregulation, could also have contributed to hepatocyte membrane stabilization under doxorubicin stress. However, this remains speculative, as Nrf2 or its downstream targets were not assessed. Alternative explanations, including methodological limitations of the TBARS assay and the timing of tissue collection (with transient oxidative or transaminase elevations possibly declining before sampling), should also be considered. Taken together, these paradoxical results highlight the importance of cautious interpretation and the need for future studies incorporating direct Nrf2 assessment and complementary oxidative stress assays.

DAPA most robustly reduced ALT/AST, nearly normalizing values, in line with its hepatoprotective profile and reports of transaminase improvement and safety in steatosis/cirrhosis [18,50], as well as experimental membrane-stabilizing/anti-inflammatory actions [10,24]. TMZ did not lower transaminases and AST increased, plausibly through mitochondrial AST modulation. Combination therapy did not exceed DAPA, supporting partial complementarity rather than synergy.

STZ and STZ + DOXO showed pronounced degeneration, necrosis, steatosis, and congestion; both agents mitigated these changes, and the combination yielded the lowest global injury scores. Benefits of dual therapy were most evident for steatosis and congestion, whereas biochemical endpoints showed no consistent advantage over DAPA alone—consistent with reports that DAPA limits hepatic lipid accumulation/inflammation via FoxO1 and SREBP-1c [19], and that TMZ reduces ischemia–reperfusion injury by supporting ATP production [25]. Accordingly, we refrain from claiming pharmacological synergy and interpret the dual-agent findings as partial complementarity limited predominantly to structural (histological) domains.

Immunohistochemical analysis confirmed that diabetes and doxorubicin exposure increased TNF-α, caspase-3, 8-OHdG, and TGF-β1 expression, all of which were attenuated by treatment. Dapagliflozin more effectively reduced caspase-3 and 8-OHdG levels, in parallel with its effects on ALT, whereas trimetazidine exerted greater effects on MDA, TNF-α, TGF-β, and GSH, suggesting complementary but non-overlapping mechanisms. Both agents significantly lowered TGF-β1 expression, with the combination showing the most pronounced suppression, indicating superior modulation of profibrotic signaling in this model.

In addition to these markers, TUNEL assay demonstrated a similar trend, with significantly higher apoptotic hepatocyte counts in the STZ and STZ + DOXO groups and a marked reduction following DAPA, TMZ, and especially combined therapy. This result provides histological confirmation that both agents effectively limited DNA fragmentation–mediated apoptosis, complementing caspase-3 findings. Likewise, NF-κB/p65 immunostaining showed intense nuclear and cytoplasmic expression in the STZ and STZ + DOXO groups, indicating activation of inflammatory transcriptional signaling. Both DAPA and TMZ treatment significantly reduced NF-κB/p65 immunopositivity, with the greatest attenuation observed in the combined therapy group, suggesting effective suppression of NF-κB–driven cytokine cascades.

Transforming growth factor-β (TGF-β) is a central mediator of hepatic fibrogenesis, primarily by activating hepatic stellate cells and promoting extracellular matrix deposition. Beyond its profibrotic effects, TGF-β is tightly interconnected with inflammatory signaling: proinflammatory cytokines such as TNF-α and Interleukin-1 beta (IL-1β) stimulate TGF-β expression, while TGF-β in turn enhances immune cell recruitment and cytokine release, thereby establishing a vicious cycle of inflammation and fibrosis [18,19,21,23,51]. The marked upregulation of TGF-β in the STZ and STZ + DOXO groups in our study likely reflects this dual role. Importantly, the reduction of TGF-β immunoreactivity by dapagliflozin and trimetazidine suggests that both agents can disrupt this cycle. This aligns with previous reports showing that dapagliflozin suppresses the TGF-β/Smad pathway, while trimetazidine mitigates profibrotic responses through antioxidant and antiapoptotic actions [52]. The concurrent downregulation of NF-κB/p65 in the present study supports this interpretation, since NF-κB is a critical upstream regulator of TNF-α and TGF-β signaling. Together, these findings suggest that the observed hepatoprotection involves coordinated inhibition of apoptotic (TUNEL, caspase-3) and inflammatory (NF-κB, TNF-α) pathways.

Our findings indicate that the protective effects of dapagliflozin and trimetazidine were largely complementary rather than synergistic, as each agent appeared to target distinct pathways of hepatocellular injury. These preclinical findings raise the possibility that combining agents with non-overlapping mechanisms may offer broader protection in complex injury settings, although whether this has any clinical relevance remains uncertain. However, the absence of clear synergy implies that the benefit of such a combination may lie in additive protection rather than potentiation of individual effects. Future translational studies should therefore focus on whether combination therapy yields clinically meaningful advantages—such as improved tolerance to chemotherapy or reduced progression of hepatic injury—beyond those achieved by single-agent treatment.

Our findings suggest that dapagliflozin’s hepatoprotective effects may extend beyond its known metabolic actions, but whether this has implications for chemotherapy-related liver injury requires further confirmation. These results are consistent with reports of multi-organ protection by SGLT2 inhibitors, although any clinical translation remains speculative. The addition of trimetazidine may have contributed to these protective effects, a finding that merits mechanistic follow-up, though its clinical significance cannot be inferred from this pilot study. Future studies should specifically address the modulation of molecular signaling pathways, the role of endoplasmic reticulum stress attenuation, and autophagic regulation, with validation in larger and more clinically relevant models.

This study has several limitations. First, caution is needed when extrapolating findings from animal models to clinical contexts. Immunohistochemical analyses were not validated by molecular assays such as RT-PCR or Western blotting, and only two markers each for inflammation (TNF-α, NF-κB) and apoptosis (caspase-3, TUNEL) were examined. Including a broader panel (e.g., IL-6, IL-1β, Bax, Bcl-2, cytochrome c) would provide a more comprehensive mechanistic picture. Another limitation is the use of archived tissues originally collected for a cardiotoxicity study; although dosing, duration, and animal numbers were consistent with standard hepatotoxicity models, the design and power calculations were not prospectively tailored for liver outcomes. In addition, blood glucose was measured only once after STZ induction, and longitudinal glycemic data were not collected; thus, dapagliflozin’s glucose-lowering effect remains a potential confounder. As a pilot study, no outliers were excluded, sensitivity analyses were not performed, and multiplicity corrections were applied inconsistently (Bonferroni for AST, Welch ANOVA/Tamhane’s T2 for others). Furthermore, only male rats were included, precluding assessment of sex-related differences. Although histopathological scoring was performed by a single blinded pathologist and TBARS is widely used for lipid peroxidation, both approaches have inherent limitations. Taken together, these factors highlight the need for future studies with larger, prospectively powered cohorts, inclusion of both sexes, and molecular validation of mechanistic pathways.

## 5. Conclusions

In conclusion, this study provides preliminary evidence that dapagliflozin and trimetazidine may exert complementary hepatoprotective effects in an experimental model of diabetes and doxorubicin-induced liver injury. However, the findings should be interpreted cautiously given the methodological limitations, and any clinical or mechanistic extrapolation remains speculative. Future studies with larger, prospectively designed methods, inclusion of both sexes, and molecular validation are required to substantiate these preliminary observations.

## Figures and Tables

**Table 1:** Biochemical Parameters in Liver Tissue and Serum Across Experimental Groups.

Group	MDA (TBARS) (nmol/g Tissue)	GSH (TT) (mM/g Tissue)	AST (IU/L)	ALT (IU/L)
Control (1)	62.30 ± 10.3	7.43 ± 1.0	157 ± 36	65 ± 9
STZ (2)	78.88 ± 17.2	6.51 ± 1.3	485 ± 120	201 ± 78
STZ + DOXO (3)	67.36 ± 11.5	8.14 ± 1.1	203 ± 83	104 ± 44
STZ + DOXO + TMZ (4)	55.40 ± 11.8	10.02 ± 3.0	501 ± 138	188 ± 42
STZ + DOXO + DAPA (5)	64.72 ± 11.4	7.12 ± 1.3	227 ± 63	64 ± 17
STZ + DOXO + TMZ + DAPA (6)	62.37 ± 2.7	7.23 ± 2.0	172 ± 120	92 ± 51

Abbreviations: MDA, malondialdehyde; TBARS, Thiobarbituric Acid Reactive Substances; GSH, AST, Aspartate transferase; ALT, alanine transferase; IU/L, international units per liter; glutathione; TT, total thiol; STZ, streptozotocin; DOXO, doxorubicin; TMZ, trimetazidine; DAPA, dapagliflozin. *n* = 8 rats per group.

**Table 2:** One-Way ANOVA Results for Biochemical Parameters Across Groups.

		Sum of Squares	df	Mean Square	F	*p*
MDA (nmol/g Tissue)	Between Groups	2,436,350	5	487,270	3610	0.008
	Within Groups	5,669,287	42	134,983		
	Total	8,105,637	47			
GSH (mM/g tissue)	Between Groups	60,961	5	12,192	3918	0.005
	Within Groups	130,707	42	3112		
	Total	191,668	47			
AST (IU/L)	Between Groups	1,004,933,020	5	200,986,604	20,041	<0.001
	Within Groups	421,213,822	42	10,028,901		
	Total	1,426,146,842	47			
ALT (IU/L)	Between Groups	147,763,583	5	29,552,717	13,796	<0.001
	Within Groups	89,968,423	42	2,142,105		
	Total	237,732,007	47			

Abbreviations: MDA, malondialdehyde; GSH, glutathione; AST, Aspartate transferase; ALT, alanine transferase.

**Table 3:** Summary of Effect Sizes (η^2^), Corresponding Cohen’s f Values, and Post hoc Statistical Power for ANOVA Results.

Parameter	Eta-Squared	Cohen’s-f	PostHoc-Power
MDA	0.301	0.656	0.928
GSH	0.318	0.683	0.948
AST	0.705	1.545	1.0
ALT	0.622	1.282	1.0

Note: Eta-squared (η^2^) represents the proportion of total variance explained by group differences. Cohen’s f is derived from η^2^ and represents the effect size for ANOVA. Post hoc power analysis was performed based on F-test at α = 0.05 level for 6 groups and 48 subjects in total (*n* = 8 in each group). Results with power value ≥ 0.80 are considered statistically sufficient. Abbreviations: MDA, malondialdehyde; GSH, glutathione; Aspartate transferase, AST; alanine transferase, ALT.

**Table 4:** Post hoc Pairwise Comparisons of AST Levels Between Groups Using Bonferroni Correction.

Group (I)	Group (J)	Mean Difference (I–J)	Std. Error	*p*-Value	95% CI Lower	95% CI Upper
1	2	−327.75	50.07	<0.001	−483.60	−171.90
1	4	−343.67	50.07	<0.001	−499.51	−187.82
2	3	282.31	50.07	<0.001	126.47	438.16
2	5	258.27	50.07	<0.001	102.42	414.12
3	4	−298.23	50.07	<0.001	−454.08	−142.38
4	5	274.19	50.07	<0.001	118.34	430.03
6	2	−312.73	50.07	<0.001	−468.58	−156.89
6	4	−328.65	50.07	<0.001	−484.50	−172.80

Note: Pairwise comparisons were performed using Bonferroni correction following one-way ANOVA. Only statistically significant results (*p* < 0.05) are shown. Negative values indicate that Group (I) had lower mean AST levels than Group (J). CI = Confidence Interval. Group (I) and Group (J) refer to the pairwise comparisons conducted between group means. The mean difference is calculated as: mean of Group (I) minus mean of Group (J). *n* = 8 rats per group.

**Table 5:** Histopathological liver damage scoring (LDS).

Group	Intralobular Degeneration and Focal Necrosis	Periportal Bridging Necrosis	Steatotic Hepatocytes	Vascular Congestion	LDS
Control (1)	0 (0–0)	0 (0–0)	0 (0–0)	0 (0–0)	0 (0–0)
STZ (2)	3 (2–3) ^a^	2 (1–2) ^a^	2 (2–3) ^a^	2 (2–2) ^a^	8 (8–9) ^a^
STZ + DOXO (3)	3 (2–3) ^a^	2 (2–3) ^a,b^	2 (2–3) ^a,b^	3 (2–3) ^a,h^	10 (9–11) ^a,h^
STZ + DOXO + TMZ (4)	1 (1–2) ^a–c^	1 (1–1) ^a–c^	0 (0–0) ^b,c^	0 (0–1) ^b,c^	3 (2–3) ^a–c^
STZ + DOXO + DAPA (5)	1 (1–2) ^a–c^	1 (1–1) ^a–c^	1 (0–1) ^b–e^	0 (0–1) ^b–d,f^	3 (3–4) ^a–c^
STZ + DOXO + TMZ + DAPA (6)	1 (1–1) ^a–c^	1 (0–1) ^a–c^	0 (0–1) ^b,c^	0 (0–1) ^b,c,g^	2.5 (2–3) ^a,c,i^

^a^ *p* = 0.001 versus Control Group, ^b^ *p* = 0.001 versus STZ Group, ^c^ *p* = 0.001 versus STZ + DOXO Group, ^d^ *p* = 0.012 versus Control Group, ^e^ *p* = 0.012 versus STZ + DOXO + TMZ Group, ^f^ *p* = 0.035 versus Control Group, ^g^ *p* = 0.008 versus Control Group, ^h^ *p* = 0.005 versus STZ Group, ^i^ *p* = 0.004 versus STZ Group, Pairwise comparisons were performed using the Dunn test with Bonferroni correction following a significant Kruskal–Wallis test. Abbreviations: LDS, liver damage scoring; STZ, streptozotocin; DOXO, doxorubicin; TMZ, trimetazidine; DAPA, dapagliflozin. *n* = 8 rats per group.

**Table 6:** Immunohistochemical Positivity Score Results (median-(25th–75th percentiles).

Group	Caspase-3 Positivity Score	8-OHdG Positivity Score	TNF-α Positivity Score	TGF-1β Positivity Score	TUNEL Positivity Score	NF-kβ/p65 Positivity Score
Control	0 (0–0)	0 (0–0)	0 (0–0)	0 (0–0)	0 (0–0)	0 (0–0)
STZ	2 (2–2) ^a^	2 (2–3) ^a^	2 (2–3) ^a^	2 (1–2) ^a^	2 (2–2) ^a^	2 (2–2) ^a^
STZ+ DOXO	3 (2–3) ^a^	3 (2–3) ^a^	3 (2–3) ^a^	2 (2–3) ^a^	2 (2–3) ^a^	3 (2–3) ^a^
STZ + DOXO + DAPA	1 (0–1) ^a–c^	1 (1–1) ^a–c^	1 (1–2) ^a–c^	1 (0–1) ^b,c,j^	1 (0–1) ^a–c^	1 (1–1) ^a–c^
STZ + DOXO + TMZ	1 (0–1) ^b–d^	1 (1–2) ^b–d^	2 (2–3) ^b,f,g,h^	2 (1–2) ^a,k^	1 (0–2) ^a–c^	1 (1–2) ^a,c,m^
STZ + DOXO + DAPA + TMZ	1 (1–1) ^a–c^	2 (1–2) ^a–c,e^	2 (1–2) ^a,c,i^	0.5 (0–1) ^c,l^	1 (0–1) ^a–c^	1 (0–1) ^b,c^

^a^ *p* = 0.001 versus to Control Group, ^b^ *p* = 0.001 versus to STZ Group, ^c^ *p* = 0.001 versus to STZ+Doxo Group, ^d^ *p* = 0.03 versus to Control Group, ^e^ *p* = 0.012 versus to STZ + DOXO+DAPA Group, ^f^ *p* = 0.044 versus to Control Group, ^g^ *p* = 0.023 versus to STZ + DOXO Group, ^h^ *p* = 0.001 versus to STZ+DOXO+DAPA Group, ^i^ *p* = 0.039 versus to STZ + DOXO + DAPA Group, ^j^ *p* = 0.026 versus to Control Group, ^k^ *p* = 0.013 versus to STZ + DOXO + DAPA Group, ^l^ *p* = 0.001 versus to STZ + DOXO + TMZ Group, ^m^ *p* = 0.006 versus to STZ Group. Pairwise comparisons were performed using the Dunn test with Bonferroni correction following a significant Kruskal–Wallis test. For each marker, liver sections from eight rats per group (total N = 48) were analyzed. Each rat contributed five sections (8 × 6 groups × 5 = 240 sections in total), and 10 non-overlapping microscopic fields per section were evaluated at 40× magnification. Abbreviations: 8-OHdG, 8-hydroxydeoxyguanosine; TUNEL, Terminal deoxynucleotidyl transferase dUTP nick end labeling; NF-kβ/p65, nuclear factor kappa B; TNF-α, Tumor necrosis factor alpha; TGF-1β, Transforming growth factor beta-1; STZ, streptozotocin; DOXO, doxorubicin; TMZ, trimetazidine; DAPA, dapagliflozin. *n* = 8 rats per group.

## Data Availability

Original data supporting the findings of this study are available. No copyright permissions are required for the figures in this study.
